# Phage-based delivery of CRISPR-associated transposases for targeted bacterial editing

**DOI:** 10.1073/pnas.2504853122

**Published:** 2025-07-25

**Authors:** Avery Roberts, Benjamin A. Adler, Brady F. Cress, Jennifer A. Doudna, Rodolphe Barrangou

**Affiliations:** ^a^Department of Food, Bioprocessing and Nutrition Sciences, North Carolina State University, Raleigh, NC 27695; ^b^Innovative Genomics Institute, University of California, Berkeley, CA 94720

**Keywords:** genome editing, CRISPR, Cas, phage, transposase

## Abstract

Phage λ, a well-characterized temperate phage, has been recently leveraged for bacterial genome editing by selectively delivering base editors into targeted bacterial species. We extend this concept by engineering phage λ to deliver CRISPR-guided transposases, accomplishing large insertions and targeted gene disruptions. To achieve this, we engineered phage λ using homologous recombination paired with Cas13a-based counterselection for precise phage modifications. Initially, we established the utility of Cas13a in phage λ by conducting minimal recoding edits, deletions, and insertions. Subsequently, we scaled up the engineering to embed the comprehensive DNA-editing CRISPR-Cas transposase (DART) system within the phage genome, creating λ-DART phages. These modified λ-DART phages were then employed to infect *Escherichia coli*, generating CRISPR RNA-guided transposition events in the host genome. Applying our engineered λ-DART phages to monocultures and a mixed bacterial community comprising three genera led to efficient, precise, and specific gene knockouts and insertions in the targeted *E. coli* cells, achieving editing efficiencies surpassing 50% of the population. This research enhances phage-mediated genome editing by enabling efficient in situ gene integrations in bacteria, offering an avenue for further application in microbial community contexts. This scalable method enables flexible microbial genome editing in situ to manipulate the function and composition of diverse ecosystems.

## Significance

Diverse microbial communities inhabit and impact most ecosystems on planet earth. While CRISPR-based technologies have enabled flexible bacterial and phage genome editing in vitro over the past decade, there is a need for novel delivery technologies to manipulate bacteria in situ. Here, we engineer phage λ with CRISPR-associated transposases to enable flexible bacterial genome manipulation, including in a mixed microbial community context. This delivery modality allows for flexible bacterial genome engineering in diverse ecosystems for the functional and compositional manipulation of microbiomes.

Microbial communities are present within diverse environments on Earth and are composed of numerous interacting microorganisms, many of which have not been successfully cultivated in the laboratory (1, 2). Yet, even when individual members of a microbial community can be cultured, studying them in isolation removes them from their native context, preventing investigations of key community dynamics and interspecies interactions (3). While in situ manipulation enables the study of microbial communities, current methods are often restricted to the removal of existing target species or introduction of nonnative or pre-engineered species, limiting the scope of possible investigations (4). For example, probiotic strains added to a community may confer health benefits by introducing new metabolic potential and outcompeting pathogenic bacteria (5, 6). Antibiotics, too, may be applied to selectively modify or perturb microbial communities based on existing or developed resistances (7). Bacteriophages (phages), the viruses that infect bacteria, have also been used to selectively perturb microbial communities (8–10). Phages generally possess species- or strain-level specificity in their infection patterns and offer an attractive avenue for targeted nucleic acid and nanoparticle delivery (11, 12). In line with their natural predation of bacteria, phages have been engineered to deliver transiently expressed genes, CRISPR arrays, or CRISPR-Cas nucleases to enhance their lethality as potent antimicrobials (13–15). These strategies, however, do not allow for precise genetic modifications of specific community members, such as producing stable in situ gene knockouts or targeted DNA integrations in the chromosome of a single strain of the community. Consequently, the development of sophisticated in situ genome editing tools is crucial for precisely manipulating the genetic makeup of members within complex microbial communities, enabling functional studies within their natural context.

As naturally occurring agents of genetic exchange in bacterial communities, phages are well suited for in situ genome editing delivery vehicles. Indeed, phages have already been genetically engineered to carry out targeted genetic modifications in the hosts they infect, shifting their role from bactericidal agents to programmable tools for precise genome editing (16, 17). CRISPR-Cas systems, the adaptive immune systems of prokaryotes that defend against invaders like phages, have served as the basis for phage engineering tools and phage-deployed genome editors alike (16, 18–20). To date, base editors (a fusion of a nickase Cas9 and a deaminase) have been deployed in phage-mediated host genome editing efforts for introducing point mutations within specific bases at a target site, albeit constrained by the need for a well-positioned protospacer adjacent motif (PAM) and a small active editing window (16, 17, 21). Furthermore, in the context of microbiome editing, base editing outcomes are generally limited to loss-of-function mutations or random mutagenesis, both of which may be readily reverted given sufficient selective pressure acting on the population. Though base editors are effective in some scenarios despite their relatively limited scope of possible genetic changes, methods for large-scale edits, such as site-specific integrases and transposons, are capable of inserting entire genes but lack the targeting flexibility that CRISPR-guided tools provide (22, 23). To circumvent these constraints, we employed the DNA-editing all-in-one RNA-guided CRISPR-Cas transposase (DART) system that incorporates the CRISPR, gene, and transposon components of a type I-F CRISPR-associated transposon (CAST) within a single vector (24, 25). Type I-F CASTs carry out CRISPR RNA-guided DNA transposition by integrating a transposon approximately 49 base pairs (bp) downstream from a target site, generally with a minimal 5′-CN-3′ PAM requirement, enabling relatively diverse target site selection (26–28). In addition, the ability of these systems to mobilize kilobase-scale genetic payloads can facilitate simultaneous gene inactivation and gene delivery when the donor transposon is directed to integrate within an existing gene (24, 26). DART, initially developed as a nonreplicative plasmid-based system, and INTEGRATE, a replicative plasmid-based system, were both employed for conjugative delivery and targeted editing within microbial communities at the strain and locus level, leading to the isolation of edited strains (24, 26). Replicative plasmid CAST systems, while effective, may lead to unintended off-target integration events over time and introduce plasmid backbones that remain within the population and express additional genes not involved in the integration process. Given the demonstration of DART as an effective gene editing system in microbial communities despite its intentionally limited persistence within a population, we sought to develop an alternative method for delivering DART for community editing. To overcome the limitations of conjugation-based community editing, such as the requirement for donor-recipient compatibility, we turned to phages as an avenue for DART delivery. A phage-based delivery approach may offer several advantages over conjugation: phage–host specificity patterns may afford a relatively narrow target species or strain range that enables expanded target site selection, and phages may provide access to spatial niches not efficiently accessible by donor bacteria. In this work, we chose to focus on the well-characterized *Escherichia coli* phage lambda (λ) to test the feasibility of this approach (29). Drawing from the previous use of phage λ to express a base editor from a prophage state, our goal was to engineer phage λ to encode and deliver the DART system for phage-mediated host genome editing (16). Critically, we eliminated an avenue for persistent phage maintenance by engineering nonlysogenic, DART-encoding phage which lack components essential for lysogeny.

**Fig. 1.**
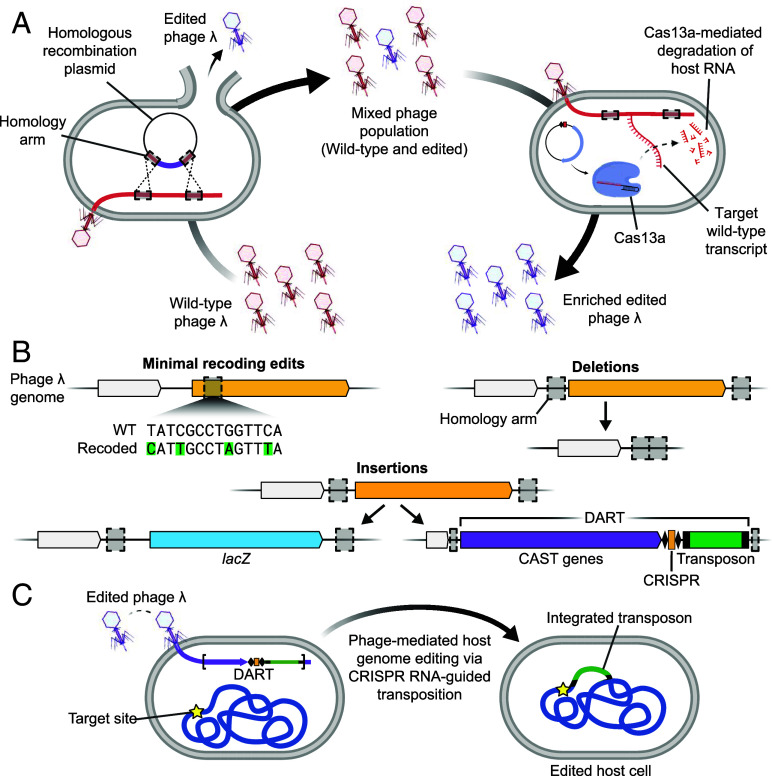
Phage λ engineering and application overview. (*A*) Graphical overview of the homologous recombination-based editing and Cas13a-mediated counterselection for enrichment of edited phage λ. Strains containing editing plasmids with regions of homology to the λ genome facilitate homologous recombination events during phage infection. Edited phages can be enriched from these lysates through Cas13a counterselection by targeting the transcript of a locus missing from edited phages. (*B*) Graphical overview of the types of edits achieved in the phage λ genome; minimal nucleotide-level edits, deletions, and insertions. (*C*) Application of edited phage containing DART, the CAST system, for phage-mediated host genome editing through CRISPR RNA-guided transposition.

In pursuit of a robust phage engineering platform, we turned to Cas13a, a CRISPR RNA-guided, RNA-targeting nuclease that provides robust immunity to phage infection (30, 31). Following target RNA binding and *cis*-cleavage, Cas13a *trans*-cleavage of host RNA molecules induces cellular dormancy that halts the phage infection cycle (30). For phage engineering, this Cas13a activity has been harnessed as a counterselection strategy following homologous recombination, both with and without the use of a selectable marker gene to isolate edited phage (31, 32). We adopted the markerless Cas13a counterselection strategy for use with phage λ, noting that Cas13a has been shown to provide immunity toward this specific phage (31) (Fig. 1*A*). As our ultimate goal was to replace over 10 kb of the λ phage genome, we first validated our phage engineering scheme through a series of smaller deletions and insertions (Fig. 1*B*). Following this initial success, the strategy was employed to incorporate the entire DART system into the λ phage genome in a single recombination event. Finally, our investigations confirm the utility of phages engineered to deliver DART for precise genome editing within a strain in single- and mixed-species cultures (Fig. 1*C*). These experimental outcomes, informed by adjustments in promoter strength, multiplicity of infection (MOI), and incubation periods, underscore the significance of these variables for enhancing editing efficiency.

## Results

### Assessment of Amber-Suppressor Hosts and λ Phage Mutants for Controlled Infection.

Our first aim was to select a delivery scheme that would allow phages to serve as delivery vectors without significantly compromising the viability of the host bacteria or their population dynamics. We also sought a system that could be adapted for both phage engineering and bacterial genome editing assays. To this end, we investigated well-studied temperate phage λ as a potential delivery chassis for controlled infection. In addition to its well-studied gene content, individual λ have been employed in biotechnological contexts to leverage λ’s viral capacity to transduce DNA while controlling viral spread during the λ life cycle. In particular, we were interested in the Sam7 mutation, an amber mutation in the λ phage cell lysis gene *S* (33–36), constraining phage lysis and viral spread to a permissive amber-suppressor host (33). In addition, we also considered the cI857 mutation which confers thermolability to the repressor protein crucial for initiating and maintaining lysogeny (36). At 30 °C, the repressor remains stable and functional; however, when the temperature is elevated to 37 °C, its stability decreases and induces lytic activity. To evaluate the feasibility of this system, we conducted phage infection assays on both wild-type (WT) *E. coli* BW25113 and amber-suppressor strain LE392MP using two λ phage variants: λ cI857 *bor::kan* and λ cI857 Sam7 (*SI Appendix*, Fig. S1*A*). Infections conducted at 37 °C revealed that the λ cI857 *bor::kan* phage caused a pronounced decline in population size across various MOIs for both strains. In contrast, the λ cI857 Sam7 phage inflicted comparable population declines in the amber-suppressor host but not in the WT BW25113 strain. A clear correlation was observed between the timing of the population decline and the MOIs: Higher MOIs resulted in rapid and steep population decreases, whereas lower MOIs led to more protracted and subtle declines in growth. Moreover, approximately 8 h postinfection, populations infected at the highest MOIs began to rebound, a pattern that emerged later in infections with lower MOIs. Notably, infections of the BW25113 strain with the Sam7-containing phage did not exhibit any significant disruptions in OD relative to the uninfected control. Based on these outcomes, we focused on the cI857 and Sam7 mutations for a flexible temperature- and genotype-based phage engineering and host genome editing strategy (*SI Appendix*, Fig. S1*B*). Complementing the temperature-sensitive regulation of cI857, the Sam7 mutation provides a secondary layer of control to minimize phage-induced lysis in the WT hosts targeted for editing.

### Cas13a-Based λ Phage Genome Editing.

**Fig. 2.**
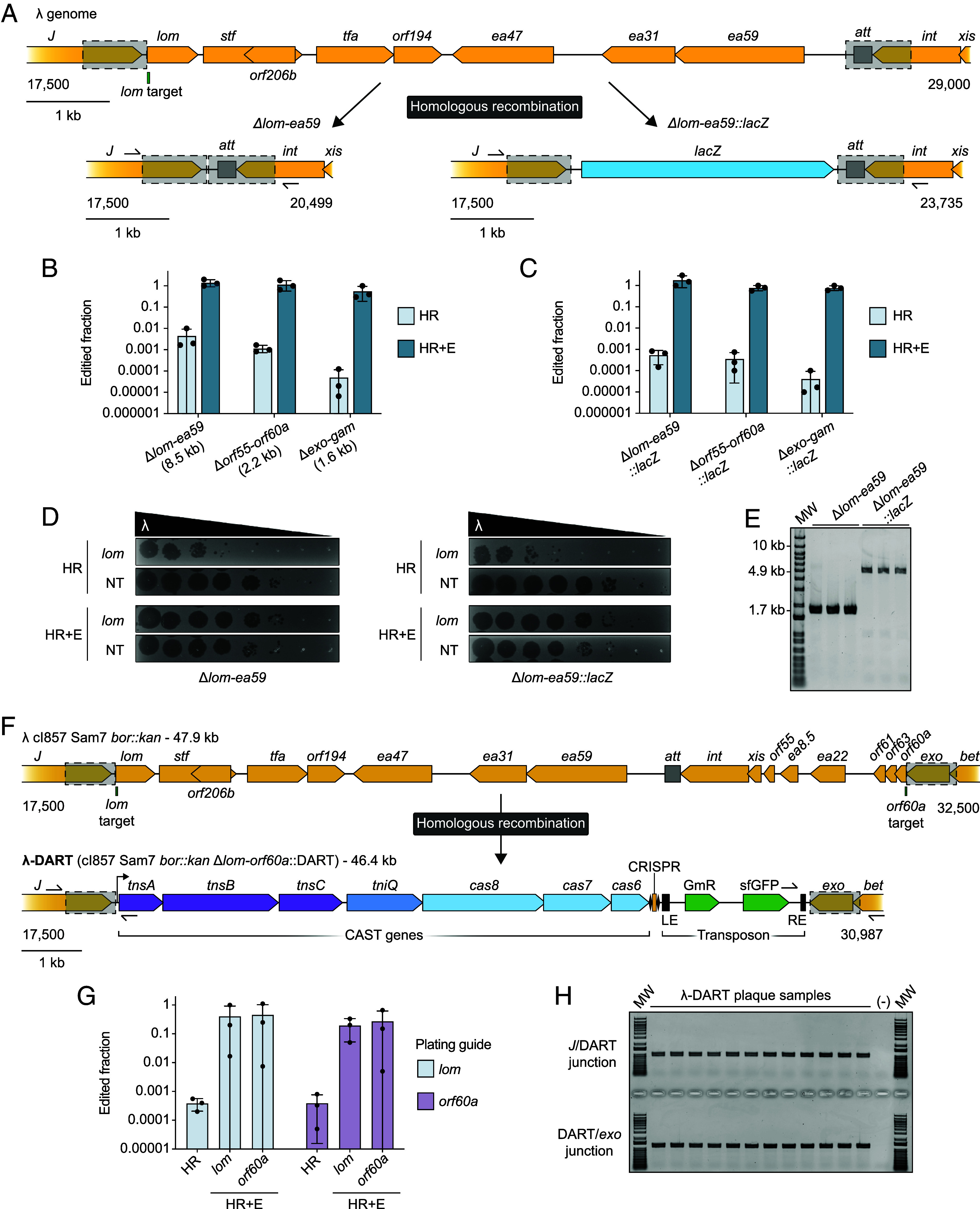
Cas13a counterselection enables diverse editing outcomes in the phage λ genome. (*A*) Overview of a nonessential region (*lom*-*ea59*) of the phage λ genome and deletion (Δ*lom-ea59*) and insertion (Δ*lom-ea59::lacZ*) editing outcomes. Homology arms for homologous recombination (HR) editing are labeled as gray boxes with dashed line borders. The Cas13a target transcript is labeled within the *lom* gene, and confirmatory PCR primers positions are shown positioned outside of the homology arms. (*B*) Edited fraction data determined after HR and subsequent enrichment (HR+E) of the intended deletion edits. The size of the deleted region is listed in parenthesis. (*C*) Edited fraction data determined after homologous recombination (HR) and subsequent enrichment (HR+E) of the intended *lacZ* insertion edits. (*D*) Phage titers were determined after the HR and HR+E steps of the editing and enrichment process for edits Δ*lom-ea59* and Δ*lom-ea59::lacZ*. Phages were spotted on strains expressing Cas13a and a counterselection (*lom*) or a nontargeting (NT), negative control guide with homology to RFP. (*E*) Confirmatory PCR results from plaques made by phage enriched for the Δlom-ea59 or Δlom-ea59::lacZ edit. For reference, the corresponding WT amplicon is predicted to be 10.2 kb. (*F*) Overview of the editing strategy for replacing a nonessential region (*lom*-*orf60a*) with the DNA-editing all-in-one RNA-guided CRISPR-Cas transposase (DART) system. Homology arms for HR editing are labeled as gray boxes with dashed line borders. The two tested Cas13a target transcripts are labeled within the *lom* and *orf60a* genes. Confirmatory PCR primers positions are shown flanking the homology arms. (*G*) Edited fraction data determined after HR and HR+E for the Δ*lom-orf60a::*DART edit. Two Cas13a guides (*lom* and *orf60a*) were individually tested for enrichment and plating for phage titering. (*H*) Confirmatory PCR results from plaques made by phage enriched for the Δ*lom-orf60a::*DART edit. Amplicons were generated using unbiased primers and amplified across both homology arm junctions. For parts *B*, *C*, and *G*, shown are the mean, SD, and individual data points from *n* = 3 biological replicates.

Recent research has explored the effectiveness of Cas13a in phage engineering, both with and without selectable markers during the editing workflow (31, 32). In this study, we applied methods from prior research that couple homologous recombination for phage editing with subsequent Cas13a-mediated counterselection to select for modified phages (31). While Cas13a demonstrated immunity against λ phage infection, λ was not among the phages edited with Cas13a-mediated methods. We sought to generate genome modifications similar to those previously obtained through Cas13a counterselection with other phages. Initially, we introduced the Sam7 mutation into λ cI857 *bor::kan* via homologous recombination (*SI Appendix*, Fig. S2*A*) and Cas13a-based enrichment (*SI Appendix*, Fig. S2*B*). Although edited phages were not observed following the homologous recombination editing step, they were successfully enriched during counterselection (*SI Appendix*, Fig. S2*C*). We confirmed the edit by PCR and sequencing, which verified the introduction of all seven base pair changes, including the Sam7 mutation alongside synonymous mutations to facilitate Cas13a counterselection, resulting in λ cI857 Sam7 *bor::kan* (*SI Appendix*, Fig. S2*D*). We then utilized the Sam7 phage in a series of experiments to produce deletions of various sizes within the nonessential regions of the λ phage genome, resembling the Cas13a-based phage genome deletions described in previous work (31). In parallel, to extend the utility of Cas13a phage engineering, we explored the potential for sizable insertions by attempting to incorporate a 3.2-kb *lacZ* cassette at the same loci (Fig. 2*A*).

For deletions, we selected nonessential regions of the λ genome, spanning approximately 1 kb to 8.5 kb in size, based on previous gene essentiality determination (29). During the editing step, strains carrying the editing plasmids exhibited comparable growth over time during infection (*SI Appendix*, Fig. S3*A*). Lysates from the initial editing step were used to infect a counterselection strain that expressed Cas13a equipped with a guide validated to confer immunity by targeting a site that was removed during the editing process. The growth dynamics of this step often, but not exclusively, suggested the presence of an enriched population of edited phage when compared to the negative control reactions that used a guide targeting an absent red fluorescent protein (RFP) gene (*SI Appendix*, Fig. S3*B*). We observed variable editing penetrance following the editing (HR) step alone, however, all deletions were fully enriched by Cas13a counterselection (HR+E) (Fig. 2*B*). A comparable trend was found for the editing penetrance for insertions at these same loci; the replacement of the 8.5 kb *lom-ea59*, 2.2 kb *orf55-orf60a*, and 1.6 kb *exo-gam* regions with the 3.2 kb *lacZ* cassette also exhibited variable penetrance after HR, but was consistently enriched during the HR+E step (Fig. 2*C*). No clear relationship was apparent between the deletion size and the editing penetrance after HR, suggesting that the size of a deletion is likely one of many factors influencing observed editing penetrance and the rate of homologous recombination in this context. The plaques produced by phages with the *lacZ* replacement were indistinguishable from those formed by phages with deletions, displaying typical morphology (Fig. 2*D*). We performed unbiased PCR on DNA from these phage plaques, followed by Sanger sequencing, to confirm the expected genotypes for deletion and insertion edits (Fig. 2*E*). These findings affirm that the Cas13a-mediated phage engineering strategy is effective for generating both minimal recoding edits and more substantial deletions and insertions within the λ phage genome.

### Incorporating a CAST System in the λ Phage Genome.

Following the successful kilobase-scale insertions in the λ phage genome, our next objective was to incorporate an all-inclusive 10.8-kb CAST system (DART) for later use in targeted transposon integration into a bacterial host genome (24). We extended the largest editing locus previously tested, the 8.5 kb *lom-ea59* region, to include *lom-orf60a* for a total 12.3-kb region of nonessential genes (29) (Fig. 2*F*). The *attP* site and integrase gene, both essential for phage integration into the host genome, are also included in this 12.3-kb region to prevent lysogenization during phage-mediated genome editing experiments (37). For this large-scale edit, we individually tested all pairwise combinations of two Cas13a enrichment guides, targeting transcripts from either *lom* or *orf60a*, for the liquid and follow-up plaque assay portions of the enrichment step (Fig. 2*G*). Performing edits with the λ cI857 Sam7 *bor::kan* background, the counterselection conducted by both guides yielded comparable results and edited phage plaques were successfully isolated from the HR step alone and HR+E. The bacterial growth over time observed during infection of these editing strains was similar to that seen from the previous deletions and insertions (*SI Appendix*, Fig. S4*A*), however the counterselection strains show no obvious signs of an edited phage population being actively enriched during infection by the HR lysates (*SI Appendix*, Fig. S4*B*). Of note, it was observed that the phage titers were considerably reduced after the enrichment step relative to previous experiments. Successful incorporation of the DART element into the phage genome was confirmed by PCR analysis of these edited phage plaques, as demonstrated by positive amplification across the boundaries of the new DART locus in all screened plaques (Fig. 2*H*). We repeated these methods to produce phages containing DART with various spacers and promoters to drive DART expression. We refer to these phages as λ-DART phages (λ cI857 Sam7 *bor::kan* Δ*lom-orf60a*::DART).

### Host Genome Editing Via a Phage-Delivered CRISPR-Associated Transposase.

**Fig. 3.**
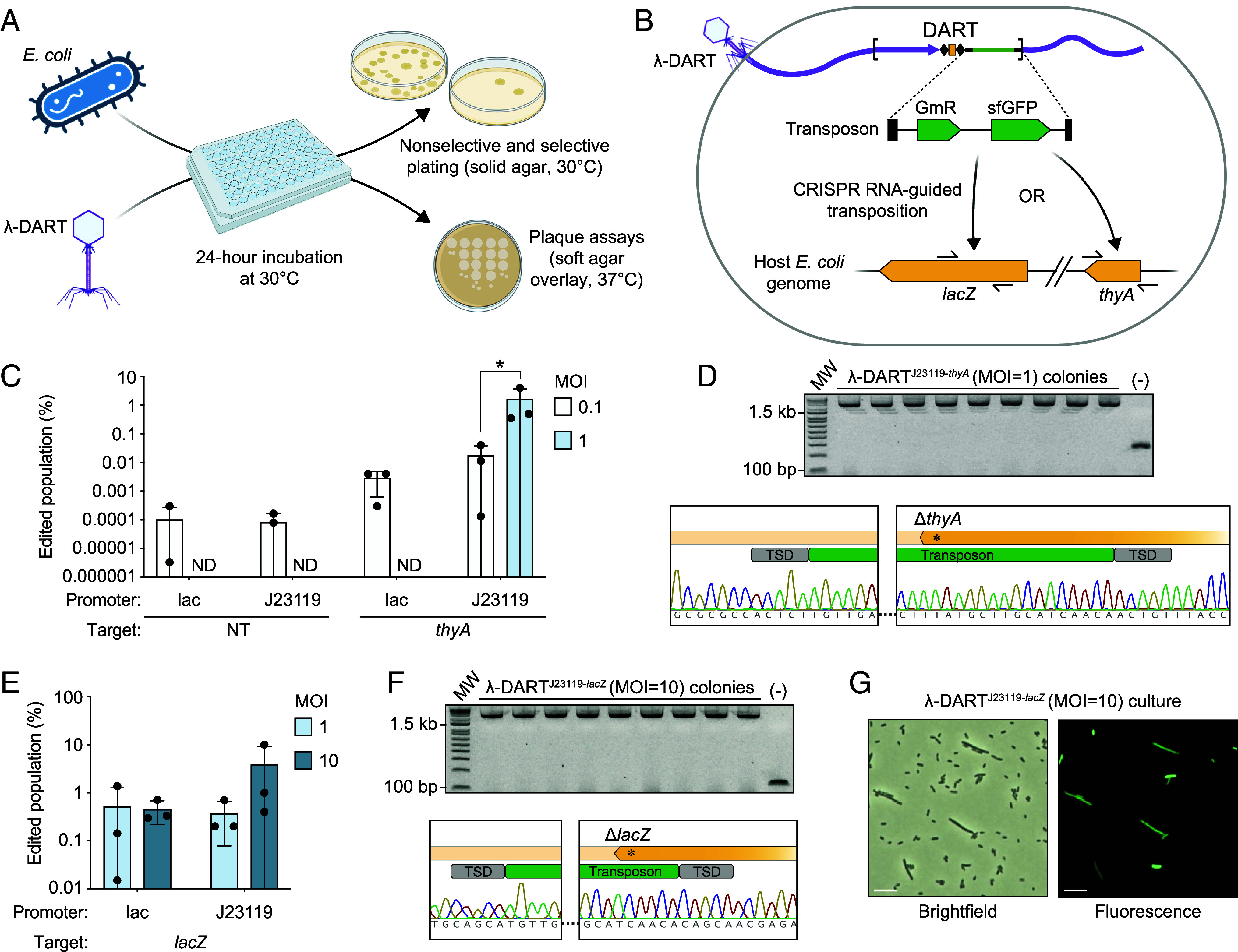
Phage-delivered DART enables targeted genome editing integration events. (*A*) Overview of the phage-mediated host genome editing assay. Edited λ-DART phage are mixed with *E. coli* host cells, incubated, then the mixtures were collected and used with solid agar plates for enumerating and isolating bacterial colonies and soft agar overlay for phage plaque enumeration and isolation. (*B*) Overview of the host genome editing strategy following λ-DART infection. DART components are expressed and facilitate CRISPR RNA-guided transposition of the donor transposon to a *lacZ* or *thyA* target site. The transposon contains gentamicin resistance and GFP reporter genes. (*C*) Editing results for hosts infected at an MOI or 0.1 or 1 by λ-DART phages containing a nontargeting or *thyA*-targeting guide with DART components driven by a lac or J23119 promoter. (*D*) Confirmatory PCR results for integration of the 2.2-kb at the *thyA* target site. Associated Sanger sequencing trace data are shown below, highlighting the *thyA* knockout. Gray rectangles labeled “TSD” correspond to expected target-site duplication events. (*E*) Editing results for hosts infected at an MOI or 1 or 10 by λ-DART phages with a lac or J23119 DART promoter and a *lacZ*-targeting guide. (*F*) Confirmatory PCR results for integrating the 2.2-kb transposon at the *lacZ* target site. (*G*) Brightfield and fluorescence (GFP) microscopy images of cells infected at an MOI of 10 by λ-DART phages with a J23119-driven DART expressing a *lacZ*-targeting guide. (Scale bar represents 10 μm.) For parts *C* and *E*, shown are the mean, SD, and individual data points from *n* = 3 biological replicates, and statistical significance is denoted as *P ≤ 0.0332; ND = not detected.

Following successful incorporation of the DART system into the λ phage genome, our next goal was to utilize λ-DART phages for host genome editing. Typically, λ-DART phages were combined with host cells, incubated for 24 h at 30 °C, and then subjected to both nonselective and selective plating, as well as plaque assays (Fig. 3*A*). This process was designed to transfer and express the DART system in the host, initiating CRISPR RNA-guided transposition and mobilizing the transposon from the phage genome to a target site in the host genome (Fig. 3*B*). In initial experiments, λ-DART phages carrying either a nontargeting guide or a *thyA*-targeting guide, under the control of either a lac or J23119 promoter, were used to infect *E. coli* MDS42 cells at an MOI of 0.1 or 1. Using medium selective for the *thyA* knockout, we observed that λ-DART phages with *thyA*-targeting guides enabled transposon integration at the intended site (Fig. 3*C*). A low proportion of background colony growth was noted in uninfected samples and those infected with nontargeting λ-DART phages, indicating that the selection process was generally, but not entirely, specific. However, infection with λ-DART phages carrying lac- or J23119-driven DART with a *thyA*-targeting guide resulted in higher proportions of cells on selective medium. Notably, samples infected by λ-DART phages carrying a *J23119* promoter and a *thyA*-targeting guide exhibited significantly higher editing efficiency at an MOI of 1 (approximately 1% of the population edited) than at an MOI of 0.1. The *thyA* integration event was confirmed by unbiased PCR across the integration site followed by sequencing of the resulting amplicon (Fig. 3*D*). Full genome sequencing of an integration-positive colony resulted in an assembled host chromosome (*SI Appendix*, Fig. S6*A*) and confirmed a single transposon integration event at the intended target site (*SI Appendix*, Fig. S6*B*). An extrachromosomal engineered phage genome was also observed in the assembly, though with relatively lower coverage than the bacterial chromosome, suggesting ongoing phage replication and persistence in the bacterial population (*SI Appendix*, Fig. S6*C*). To further examine the impact and activity of phage infection, we also measured the final cell count and phage titer increase of each sample following infection and incubation (*SI Appendix*, Fig. S5*A*). Following infection, there was a slight reduction in final cell count from samples infected at the higher MOI of 1 relative to the lower MOI of 0.1. Considering that the Sam7 mutation hinders cell lysis without affecting functional virion assembly, we compared the phage titers postinfection to the original phage stock titers. Interestingly, phage titers increased to up to 10^9^ PFU/mL from the initial titers of the phage-infected samples (5 × 10^4^ PFU/mL and 5 × 10^5^ PFU/mL for samples with an MOI of 0.1 and 1, respectively), confirming that these engineered phages are replicating and persisting within the infected population. The observed increases in phage titer were inversely correlated with the activity of the DART promoter, independent of MOI. This observation suggests that increased promoter activity, specifically that of the J23119 promoter, may burden host metabolism, thus limiting phage replication and endpoint titers.

Next, we shifted our approach to focus on λ-DART phages containing either a lac or J23119 promoter and a single *lacZ*-targeting guide, conducting infections at a higher MOI range of 1 to 10 with the aim of increasing host editing rates (Fig. 3*E*). Following incubation, cells were cultured on gentamicin-containing medium to select for transposon integration, revealing editing efficiencies of about 0.1 to 1% of the population under these experimental conditions. Notably, cells infected with phages using the J23119 promoter at an MOI of 10 yielded the highest editing rates, reinforcing the notion that both promoter strength and MOI are influential for host editing success. We measured final cell densities and phage titers, observing that while cell densities remained relatively stable across all conditions, there was a trend of lower phage titers from samples infected by J23119-driven λ-DART phages compared to those with the lac promoter at equal MOI (*SI Appendix*, Fig. S5*B*). As in previous experiments, we performed unbiased colony PCR and sequencing from colonies infected by *lacZ*-targeting λ-DART phages (Fig. 3*F*). Upon screening three colonies from each biological replicate, we found that every colony exhibited a distinct genotype confirming the insertion of the 2.2-kb DART transposon at the target site. This sample consistency implies that selective plating effectively isolates cells harboring an integrated transposon. Immediately following the infection period, we used microscopy to assess the culture infected with λ-DART phages with the J23119 promoter at an MOI of 10 (Fig. 3*G*). The proportion of cells observed with GFP fluorescence, derived from the *sfGFP* gene of the transposon, roughly corresponded to the ratio of edited cells as determined by selective medium plating. We note that these fluorescent cells often possessed relatively elongated morphology, potentially due to slowed division caused by the activity of other λ proteins (38).

### Phage-Mediated Host Genome Editing in a Community Context.

**Fig. 4.**
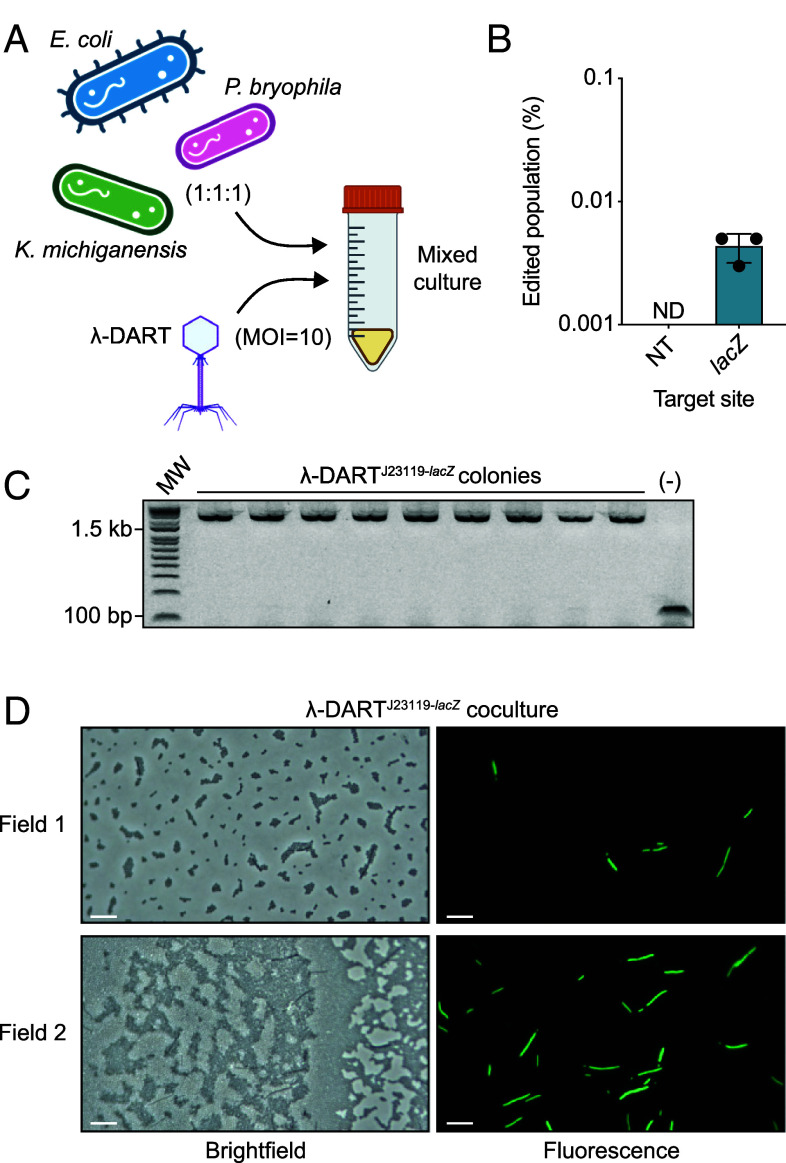
Phage-mediated host genome editing in a community context. (*A*) Graphical overview of the 3-member community editing assay. (*B*) Editing results for host cells infected at an MOI of 10 by λ-DART phages containing a nontargeting or *lacZ*-targeting guide with DART components driven by a J23119 promoter. (*C*) Confirmatory PCR results for integrating the 2.2-kb transposon at the target site from *E. coli* colonies isolated on selective plates. (*D*) Endpoint brightfield and fluorescence (GFP) microscopy images from multiple fields of a mixed culture infected at an MOI of 10 by λ-DART phages harboring a J23119 promoter and a *lacZ*-targeting guide. Field 2 shows a crowded, layered view of the mixed community culture. (Scale bar represents 10 μm.) For panel *B*, shown are the mean, SD, and individual data points from *n* = 3 biological replicates; ND = not detected.

Having achieved successful host genome editing in monoculture, we next sought to perform editing within a synthetic microbial community context, drawing inspiration from previous experiments using a phage λ-delivered base editor expressed from a prophage (16). By mixing λ-DART phages with a coculture of *K. oxytoca*, *P. bryophila*, and *E. coli* strains, we demonstrate here successful host genome editing through CRISPR RNA-guided transposition in a mixed-species community (Fig. 4*A*). Cells infected by λ-DART phage harboring a nontargeting guide resulted in no detectable editing events, while a *lacZ*-targeting guide resulted in editing between 0.001 to 0.01% of the mixed culture (Fig. 4*B*). We plated endpoint cultures on selective gentamicin-containing medium and screened colonies for integration at the *lacZ* target site and, consistent with previous observations, each screened colony possessed the expected integration genotype (Fig. 4*C*). We employed microscopy to examine samples infected by λ-DART phages targeting *lacZ* and identified fluorescent *E. coli*, often with the elongated phenotype seen in previous experiments, present within the mixed culture (Fig. 4*D*).

### Optimization of Phage-Mediate Host Genome Editing.

**Fig. 5.**
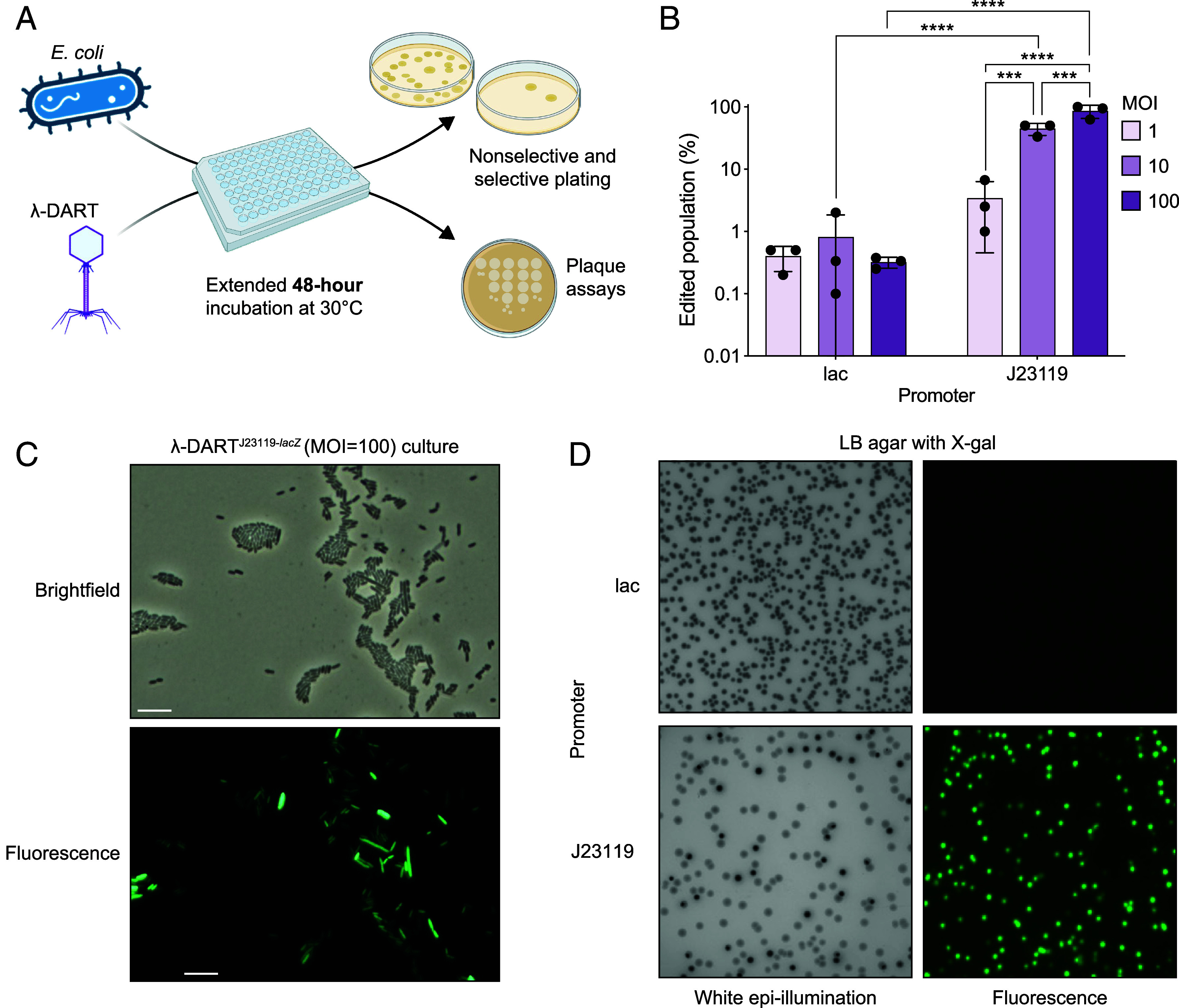
Extended incubation time increases phage-mediated host genome editing efficiency. (*A*) Overview of the phage-mediated host genome editing assay with an extended, 48-h incubation period. (*B*) Editing results for host cells infected at an MOI of 1, 10, or 100 by λ-DART phages expressing *lacZ*-targeting DART driven by a lac or J23119 promoter. (*C*) Endpoint brightfield and fluorescence (GFP) microscopy images from cells infected at an MOI of 100 by λ-DART phages harboring a J23119 promoter and a *lacZ*-targeting guide. (*D*) White epi-illumination and fluorescence (GFP) images of colonies grown on LB agar with X-gal. Plates show colonies from cells infected at an MOI of 100 by λ-DART phages harboring a lac or J23119 promoter and a *lacZ*-targeting guide, and some colonies appear dark due to blue-white screening. (Scale bar represents 10 μm.) For part *B*, shown are the mean, SD, and individual data points from *n* = 3 biological replicates, and statistical significance is denoted as ***P ≤ 0.0002 and ****P ≤ 0.0001.

In the final phase of this study, we aimed to enhance genome editing frequency in λ-DART phage-infected populations. Based on our findings, we hypothesized that increasing MOI could improve editing efficiency by delivering more DART system copies and ensuring complete cell saturation. Additionally, informed by our and others’ work on type I-F CASTs, we posited that an extended incubation period could elevate editing rates (26, 28). Therefore, we increased the liquid infection period of our assay from 24 to 48 h and used λ-DART phages with lac or J23119 promoters, a *lacZ* guide, and performed infections at an MOI of 1, 10, or 100 (Fig. 5*A*). Despite these modifications, the editing rates remained within the range of 0.1 to 1% for λ-DART phages with a lac promoter, consistent across all MOIs and comparable to those observed in prior experiments (Fig. 5*B*). However, we saw dramatic editing success from J23119-driven λ-DART phages, with significant increases in editing efficiency positively trending with MOI. Surprisingly, J23119-driven λ-DART infected at an MOI of 100 resulted in editing in over half of the population. Final cell counts and phage titers were generally consistent across samples, though phage titers matched the highest titers seen from the 24-h experiments, suggesting that extended incubation periods may allow for a saturation of phage in the population (*SI Appendix*, Fig. S5*C*). We used microscopy to examine the sample infected at an MOI of 100 with J23119-driven λ-DART phages and observed a significant proportion of the population as positive for GFP fluorescence, congruent with selective plating results (Fig. 5*C*). Unlike in previous experiments, many of these fluorescent cells did not display an elongated phenotype, perhaps due to phage clearance or cellular replication resulting in a dilution of factors contributing to the phenotype. To further assess the editing efficiency, we cultured samples infected at an MOI of 100 with λ-DART phages, driven by either lac or J23119 promoters, on nonselective medium to conduct blue/white screening and fluorescent imaging (Fig. 5*D*). Aligning with the results from selective plating, the lac-promoter-driven DART did not produce colonies with a noticeable *lacZ* knockout phenotype, nor did it induce corresponding fluorescence in colonies within this experimental setup. In contrast, cells from the J23119-promoter-driven DART sample displayed a distinct *lacZ* knockout phenotype, and these colonies exhibited strong fluorescence, suggesting a correlation between significant fluorescence and successful transposon integration. These experiments indicate that while a higher MOI has the potential to improve editing efficiency, the expression level of the DART system is a critical factor affecting the extent of genome editing.

## Discussion

We report the use of a phage–host pairing involving the temperature-sensitive λ repressor mutation cI857 and the Sam7 lysis gene mutation. This system, when applied to a host with an amber-suppressor, is useful at higher temperatures for phage engineering applications. Conversely, it facilitates host genome editing when utilized at lower temperatures with a nonsuppressor, WT host. This dual-use framework offers temperature-dependent control over the λ repressor and lysis functions, allowing for adjustable infection dynamics. Using this system, we built upon previous phage engineering work involving Cas13a counterselection strategies that do not require selectable markers (31). Although traditional λ engineering techniques are well established, we employed Cas13a counterselection due to its potential adaptability to novel phage–host systems beyond *E. coli* (39). We demonstrate that minimal edits, like introducing the Sam7 mutation while modifying a total of 7 bps, are achievable in phage λ. Additionally, we performed deletions in line with those achieved using similar methods with other *E. coli* phages, such as lytic phages T7 and T4, in previous work (31). Our largest editing locus of 8.5 kb (Δ*lom-ea59*) was enriched as successfully as the smaller tested loci for both deletion and *lacZ*-insertion edits. The 1.6 kb *exo-gam* locus had the lowest corresponding editing rates, for both the deletion and *lacZ*-insertion, and we initially hypothesized that the size of the deletion or replaced region of the native λ phage genome influences the efficiency of editing. However, we now recognize that additional variables, such as the specific genes or other elements in the section being excised from the phage genome, are also likely to affect the success of homologous recombination with the editing plasmid. Furthermore, these factors may impact the propagation of the edited phages within the diverse phage population present during the editing phase.

After confirmation of successful insertions by replacing sizable loci of the λ phage genome, we aimed to incorporate DART within the λ phage genome. To prevent the introduction and accumulation of prophage within the target host genome, we specifically included the *attP* site and integrase gene in the region to be removed from the λ phage genome during editing. To accommodate the insertion of the 10.8 kb DART system, we built upon our previously successful deletions and *lacZ* insertions within the 8.5 kb *lom-ea59* region, extending it to the 12.3 kb *lom-orf60a* region. We chose a region of this length to preserve the overall genome size due to the λ phage’s packaging constraints linked to capsid size (40). Our engineered λ-DART phages successfully met this requirement, with their genome lengths being approximately 97% that of the parental λ cI857 Sam7 *bor::kan* phage genome. Incredibly, the region of the parental phage that DART replaced is roughly a quarter of the parental phage genome (*SI Appendix*, Fig. S7). We successfully created various λ-DART phages with diverse promoters and guide RNA sequences by utilizing homologous recombination to integrate the entire DART system into the phage genome in a single step. This marks a demonstration of the Cas13a counterselection method for large-scale genetic insertions for phage engineering. These methods may be broadly applicable for phage engineering in diverse species and are complemented by recent advances in phage gene essentiality determination that inform the selection of loci amenable to removal or modification (41, 42).

In this study, we present a method of host genome editing using engineered phages that carry and introduce the DART system into the host for CRISPR RNA-guided transposition. While previous research has embedded CRISPR or CRISPR-Cas elements into phage genomes for antimicrobial purposes, and others have achieved host genome editing through base editors expressed from prophages or phage-delivered cosmids, our approach expands the potential of these efforts through species- and site-specific kilobase-scale edits (13, 14, 16, 17). We employ a phage with an engineered genome and conditional mutations to induce specific phage phenotypes, thereby facilitating host genome editing while avoiding lysogeny. We combined the temperature-sensitive transcriptional regulation of cI857, which limits lytic gene expression, with Sam7, which prevents direct phage-induced cell lysis. This combination serves as a layered approach to limit lytic outcomes in infected hosts, accounting for potential incomplete cI repression or altered regulation stemming from the inability of the engineered phage to lysogenize. While we acknowledge that the Sam7 mutation may prevent cell lysis without stopping phage replication, we propose that this may be responsible for enhanced host editing in our experiments and provides useful information for similar phage-mediated avenues for delivering CRISPR-associated transposases. If a cell releases functional virion, subsequent infection events may increase the rate of editing in the population. Similarly, if a cell contains a mix of free and encapsidated phage genomes, the natural process of cell division could spread these phage-plasmid-like elements and, over time, preferentially support editing based on the potential for a free phage genome to express DART (43). Following expression, the TniQ–Cascade complex may bind to the target site, but the phage-contained transposon must be accessible to successfully assemble the transpososome that completes transposition (44). Nevertheless, the introduction of additional amber mutations, potentially in the major capsid protein, could be explored to inhibit the assembly and possible release of functional virions. Further research is necessary to substantiate these ideas of how the rate of CRISPR RNA-guided transposition in a population is affected by the use of replicative or nonreplicative machinery given active cell division, and how quickly these elements are diluted over time. While substantial genome editing may be achievable within a population, the concurrent phage gene expression and related host fitness costs during the infection and editing process could interfere with intended experimental outcomes or downstream applications.

Our phage-mediated host editing experiments covered multiple target sites, *thyA* and *lacZ*, and achieved CRISPR RNA-guided transposition within host cells. In all cases, all screened colonies from samples infected by *thyA*- or *lacZ*-targeting λ-DART were positive for the integration event, supporting the specificity of our selective plating. Our results implicate MOI, promoter strength, and incubation time in editing efficiency. By expressing DART from a high-strength J23119 promoter, we saw a significant increase in editing efficiency relative to expression by the lac promoter during our 48-h incubation experiment. This variation in efficiency reflects findings from plasmid-based systems where promoter strength had a significant effect; however, in scenarios where the DART is expressed through a conjugative suicide vector, the influence of promoter strength on editing efficiency appears to be diminished, underscoring the importance of the editing context in determining the optimal level of expression strength (24, 26). In or beyond *E. coli*, phage-mediated DART editing outcomes may also benefit from the controlled expression of identified DART inhibitors or activators in parallel (45, 46). Coincidentally, the *exo*, *beta*, and *gam* genes of λ were found to increase editing efficiency when strongly coexpressed with the components of DART, and these genes were retained in our λ-DART phage, albeit with native transcriptional regulation (46). In our experiments, strong expression of DART enabled editing of over half of the population at the endpoint of extended assay conditions, and we show that phenotypes indicative of editing events are visible in nonselective conditions. Among these observed phenotypes, an elongated cell morphology was strongly associated with fluorescent cells at the 24-h experiment endpoint, suggesting temporarily altered cell division. This phenotype was less prevalent at 48 h, even at the highest MOI tested, potentially due to the dilution of phage factors following cell division. Furthermore, GFP fluorescence appears to be linked with editing efficiency, implying that strong GFP expression in this context is driven by chromosomally integrated transposons rather than free phage genomes, which may be undergoing both active replication and encapsidation. While phage genome replication and encapsidation represent potential caveats of the replicative phage approach employed here, alternative strategies utilizing nonreplicative vectors or relying upon temperate phage lysogeny are likely viable routes for DART delivery. The optimal choice among these methods will depend on the specific use case, as their effectiveness and adaptability across diverse strains or species, especially concerning phage engineering, require further investigation. The λ phage host range is amenable to engineering for altered *E. coli* strain specificity or promiscuity, a process that may be aided by phage–host range prediction tools (47, 48). Notably, engineered λ tail variants were incorporated into a λ cosmid system that successfully delivered base editing machinery into a *Klebsiella pneumoniae* strain (17).

We present the application of phage-mediated host genome editing using DART within a community setting. Echoing the methods of a previous study, we employed a simple community consisting of the target *E. coli* and soil-derived strains, achieving successful editing results. Our findings confirm that this phage-based system is effective in editing target microbes, facilitating the isolation and screening of target members from a community. Our approach to phage-mediated host genome editing may, given phage–host range engineering or the use of novel phage, extend to a broader range of organisms beyond *E. coli*. While this specific phage-mediated DART delivery strategy may not be suitable for all microbial systems or editing goals, we anticipate that the findings from this work will provide valuable insights for optimizing efficient phage-mediated CRISPR RNA-guided DNA integration in various community contexts. The utilization of CAST systems in microbial genome editing, as shown in this work and others, can broaden our understanding of microbial life within their native communities and the intricate interactions that define them.

## Materials and Methods

### Bacterial Strains and Growth Conditions.

*E. coli* strains BW25113, 10-beta (NEB), MDS42 (Scarab Genomics), LE392MP (Lucigen), and derivatives were used in this study. Liquid cultures were grown in lysogeny broth (LB Lennox; Difco) at 30 or 37 °C with shaking at 250 RPM. When necessary, antibiotics were supplemented in media at the following final concentrations: chloramphenicol (34 μg/mL), carbenicillin (100 μg/mL), gentamicin (20 μg/mL), and kanamycin (50 μg/mL). For *thyA* knockout selection, M9 minimal growth medium was supplemented with 0.2% casamino acids, 0.4% glucose, 100 μg/mL thymine, and 50 μg/mL trimethoprim as previously described (49). For blue/white colony screening, medium was supplemented with ChromoMax IPTG/X-Gal solution (Fisher Scientific). Media were solidified with 1.5% agar for solid culture conditions. Strains *Paraburkholderia bryophila* 376MFSha3.1 and *Klebsiella oxytoca* M5a1 were cultured at 30 °C in LB with shaking at 250 RPM. Stocks of strains were made using 15% glycerol and stored at −80 °C.

### Phage Rebooting.

Phage DNA from λ cI857 Sam7 (Thermo Scientific) was used to reboot the phage as previously described (50). Briefly, *E. coli* host LE392MP was grown overnight, pelleted, and repeatedly washed with ice-cold water and 10% glycerol to prepare electrocompetent cells. Electrocompetent cells were mixed with 1.5 μg of phage DNA and electroporated at 2 kV, 25 μF, and 200 Ω. Cells were recovered in 800 μL of SOC medium for 45 min at 37 °C with 250 RPM shaking. Following incubation, cells were pelleted and resuspended in 1 mL of 10 mM MgSO_4_. This suspension was combined with 5 mL of 0.7% molten top agar and overlaid on an LB agar plate. After solidifying, the plate was incubated at 37 °C overnight. Dozens of phage plaques were observed, and plaques were collected from the plate and phage identity was confirmed by PCR before propagation.

### Phage Handling and Propagation.

Phages were propagated by the top agar overlay and liquid scaling methods as previously described (51). All phage stocks were maintained and diluted in SM buffer (VWR). Unless noted otherwise, phages were propagated and titered on *E. coli* LE392MP. Phage λ cI857 *bor::kan* was a gift from Dr. Drew Endy. To determine phage titers, we calculated plaque-forming units per mL (PFU/mL) by 10X serial dilutions of phage and spotting 2 μL aliquots onto freshly prepared 0.7% top agar overlays.

### Plasmid Design and Construction.

Plasmids used in this study were cloned and propagated in *E. coli* 10-beta cells for further use. Plasmid DNA was extracted using a Plasmid Midi Kit (QIAGEN) and verified by long-read sequencing (Plasmidsaurus). For editing and enrichment assays, plasmids were transformed into chemically competent LE392MP cells. Phage editing plasmids contained a backbone with a p15A origin of replication and an ampicillin resistance (*ampR*) gene. Editing plasmids were constructed using the BbsI or SapI Golden Gate assembly method, with fragments being cloned into the p15A vector (52). For certain constructs, Twist Bioscience performed the cloning into the same vector. DNA fragments used for Golden Gate assembly and cloning were synthesized by Twist Bioscience or amplified from existing plasmids by PCR. The editing plasmid used to introduce the Sam7 mutation in λ cI857 *bor::kan* included a 320-bp sequence identical to the phage genome, with a seven-base modification at the Sam7 site for targeted editing. For phage λ deletions or insertions, the editing plasmids contained two homology arms, each ranging between 180 to 800 bp. Deletion plasmids had homology arms bordering a 32-bp SapI-dropout sequence, and for insertions, the homology arms surrounded the sequence intended for integration into the phage genome. All plasmids used in this study are listed in Dataset S1. Details of the homology arms of the editing plasmids are listed in Dataset S2.

Editing plasmids for DART were made in two configurations. The first configuration contained, in order, the CRISPR array, *tnsABC* and *tniQcas876* operons, and the transposon containing a gentamicin resistance cassette (GmR) and a green fluorescent protein reporter (sfGFP). Following successful phage editing involving the first configuration, a second version of DART was made by altering the design of the original DART system (24). Additional terminators were incorporated within and adjacent to DART to minimize unintended transcription across the DART transposon ends, including into the native phage genes, and from native phage genes across the right transposon end (RE). One terminator was placed between the CRISPR array and the left transposon end (LE), and a second was situated between the right transposon end (RE) and the adjacent homology arm. Further derivatives of the phage editing plasmids containing DART feature variations in spacer content and a substitution of the lac promoter with the high-strength J23119 promoter. We selected the *lacZ*-targeting spacer for its established efficacy, while the design of the *thyA*-targeting spacer was informed by recent insights into the targeting preferences of the Tn6677 CAST system, as described in the literature (25, 45).

Enrichment plasmids were derived from pBA559 and possess a p15A origin of replication, a chloramphenicol resistance cassette, a constitutively expressed crRNA BsaI-dropout region, TetR repressor gene *tetR*, and LbuCas13a expressed under TetR-pTet regulation for induction by anhydrotetracycline (aTc) (31). To clone spacers into the crRNA dropout region of pBA559, a one-cycle Golden Gate assembly reaction was performed with BsaI-HFv2 (NEB) and annealed 5′-phosphorylated oligos (IDT) with overhangs complementary to those generated in pBA559 by BsaI-HFv2. Spacers were generally designed to target the first 31 nucleotides of the transcript for a gene of interest. All DART and Cas13a guide sequences used in this study are listed in Dataset S3.

### Liquid Phage Infection Assays.

Fresh *E. coli* cultures were grown overnight at 30 °C and 250 RPM and then diluted in LB medium for a final reaction OD_600_ of 0.02 for each experiment. Wells of a 96-well plate (Corning 3,904) were each loaded with 190 μL of diluted culture, for approximately 4e6 colony-forming units (CFU), and 10 μL of phage for a final predetermined MOI. OD_600_ was measured in 5-min intervals for 16 h at 37 °C with shaking in a VANTAstar plate reader (BMG LABTECH).

### Phage Plaque Assays.

Fresh *E. coli* cultures were incubated overnight at 30 °C and 250 RPM for subsequent use with double agar overlays. These overlays consisted of equal volumes of bottom agar and a 0.7% agar overlay, the latter prepared with appropriate antibiotics and aTc when necessary. For isolated plaque assays, 100 μL of the overnight culture was combined with 5 mL of molten top agar and 25 μL of phage stock. This mixture was then poured onto an LB agar plate containing the same antibiotics. In contrast, for phage titer determination, the phage stock was serially diluted and 2 μL spots were applied to a dry top agar overlay. After the overlay set or the spots dried, plates were incubated overnight at 37 °C. Plaques were imaged using white epi-illumination on a ChemiDoc imager (Bio-Rad).

### Genome Editing of Phage λ.

The phage genome editing and enrichment strategy in this study is adapted from a recent study (31). Fresh *E. coli* cultures of strains harboring editing plasmids were incubated overnight at 30 °C and 250 RPM in LB with carbenicillin. For each phage editing assay, overnight cultures were diluted in LB for a final reaction OD_600_ of 0.02 for a total of 4e6 CFU per well. Three technical replicate wells per sample were loaded with 190 μL of diluted culture and 10 μL of phage stock containing approximately 4e4 PFU for a final MOI of 0.01. OD_600_ was measured in 5-min intervals for 7 h at 37 °C with shaking in a VANTAstar plate reader. Following incubation, replicate sample lysates were pooled and transferred to a 96-well deep well plate (Greiner). Each lysate was treated with a drop of chloroform and then incubated at room temperature for 20 min. Treated lysates were then 10X serially diluted in SM buffer before the plate was sealed with an aluminum seal. These treated lysates were stored at 4 °C until further use. We refer to these lysates as “HR” (homologous recombination) lysates as they may contain some fraction of phages edited by homologous recombination with the editing plasmid.

Fresh *E. coli* cultures of strains harboring Cas13a counterselection plasmids were incubated overnight at 30 °C and 250 RPM in LB with chloramphenicol. Enrichment assays were performed with LB medium supplemented with chloramphenicol and aTc at a final concentration of 2.5 or 5 nM. For each enrichment assay, overnight cultures were diluted in LB for a final reaction OD_600_ of 0.04 for 8e6 CFU per well. Three technical replicate wells per sample were loaded with 190 μL of diluted culture and 10 μL of a 1:10 HR phage lysate dilution. Here, we use each 1:10 HR phage lysate dilution for a reaction with a negative control guide (RFP) or a guide targeting phage λ at a locus missing from the edited phage. OD_600_ was measured in 5-min intervals for 16 h at 37 °C with shaking in a VANTAstar plate reader. As before, replicate samples treated with chloroform, diluted, and stored at 4 °C until further use. These lysates, potentially enriched with phages edited via homologous recombination, are designated as “HR+E” lysates.

Phage genome editing penetrance determination was carried out as previously described (31). Briefly, serially diluted lysates from both HR and HR+E lysates were spotted on agar overlays with strains expressing Cas13a and a negative control guide (RFP) or an enrichment guide for counterselection. For each edit, we determined the editing penetrance as the proportion of PFU on the enrichment guide strain compared to the negative control strain. This represents the proportion of the population that escapes targeting via the replacement of the target locus through homologous recombination with the editing plasmid.

To confirm phage editing, we performed confirmatory PCRs using DNA extracted from plaques of putative-edited phages. After determining editing penetrance, we performed plaque assays to isolate plaques of putatively edited phages. Plugs from these plaques were incubated in 50 μL of SM buffer at room temperature for 1 h. Subsequently, we transferred part of this mixture to PCR tubes and boiled it at 100 °C for 10 min. We then used 10 μL of the boiled sample as a template in a PCR. These PCRs used 2× Q5 Master Mix (NEB) and primers that annealed outside the homology arm regions used in the editing plasmids. This strategy prevented false positives by amplifying any carryover phage editing plasmid DNA. PCR products were visualized on 1% agarose gels with ethidium bromide alongside a 1 Kb Plus DNA Ladder (Invitrogen) and imaged using a ChemiDoc imager. Phages positive for the intended edit were passaged twice on the enrichment strain for purity before further use.

Select parental and derivative phage genomes were aligned and visualized using the LoVis4u Python package (53). Phages used or generated in this study are listed in Dataset S4. Oligos used in this study are listed in Dataset S5.

### Host Genome Editing Assays Using Engineered λ Phage.

Host genome editing assays involved *E. coli* strain MDS42 infection with λ phages engineered to contain DART. For each experiment, an overnight culture of MDS42 was incubated overnight at 30 °C with shaking. The culture was diluted in LB medium based on OD_600_ for 1e5 CFU per well. In a 96-well plate, three technical replicate wells per sample were filled with 100 μL of diluted culture and 100 μL of phage stock for a predetermined MOI. The plate was incubated at 30 °C for 24 or 48 h with shaking in a VANTAstar plate reader with OD_600_ measurements at 15-min intervals. Following incubation, samples were pooled in a 96-well deep well plate and 10X serially diluted in LB medium. Additionally, 100 μL of each undiluted sample was transferred to a separate 96-well deep well plate, treated with chloroform, and 10× serially diluted in SM buffer for phage titer determination. Cell dilutions were plated on nonselective (LB) or selective (LB with gentamicin; M9 medium with thymine and trimethoprim) agar plates in 5 μL spots. In some cases, 50 μL of diluted samples were plated on individual agar plates for isolated colonies. Plates were incubated at 30 °C for 24 to 48 h. We note that the *thyA* knockout phenotype resulted in colonies with slower growth rates. The edited population was determined as the proportion of cells on the selective plates compared to the nonselective plates. Statistical significance was determined using two-way ANOVA tests with Tukey’s post hoc test.

We performed confirmatory colony PCRs using template DNA from isolated colonies, 2× Q5 Master Mix, and primers that flank the integration site for unbiased detection of WT or edited genotypes. After PCR confirmation of integration, a *thyA*-knockout colony was picked and grown overnight at 30 °C with shaking in LB medium supplemented with gentamicin. Briefly, the overnight culture was pelleted, genomic DNA was extracted and sequenced with ONT long-reads, sequencing reads were assembled with Flye, and the assembly was annotated using Bakta (54, 55) (Plasmidsaurus). To determine coverage, reads at least 2.5 kb in length were mapped back to the assembled bacterial and phage sequences using the Minimap2 mapper in Geneious Prime with the setting of “Map multiple best matches: To none” (56) (https://www.geneious.com). We note this setting resulted in the discarding of some transposon-encoding reads given that the transposon may map to either the phage or edited host genome. Genomic loci were visualized above the coverage plots by using the DNA Features Viewer Python library (57).

For coculture experiments, 1e5 CFU each of *E. coli* MDS42, *P. bryophila* 376MFSha3.1, and *K. oxytoca* M5a1 were present in 250 μL total of LB medium and mixed with 250 μL of phage stock for predetermined MOIs. Phage-cell mixtures were transferred to conical tubes and incubated at 30 °C with 250 RPM shaking for 24 h. Samples were then serially diluted and plated for isolated colonies on nonselective and selective plates.

Cells from endpoint culture samples were imaged by aliquoting a drop of culture onto a 1% agarose pad. These samples were examined using an upright Nikon Eclipse E600 microscope equipped with a pE-300 (CoolLED) illumination system for fluorescence.

## Supplementary Material

Appendix 01 (PDF)

Dataset S01 (XLSX)

Dataset S02 (XLSX)

Dataset S03 (XLSX)

Dataset S04 (XLSX)

Dataset S05 (XLSX)

## Data Availability

All study data are included in the article and/or supporting information.
