# Supplementary material for: Phage-based delivery of CRISPR-associated transposases for targeted bacterial editing
Source: Proc Natl Acad Sci U S A. 2025 Jul 25;122(30):e2504853122. doi: 10.1073/pnas.2504853122 (PMC12318184; doi:10.1073/pnas.2504853122)
Supplement: Supplementary file 1 — Appendix 01 (PDF) [file pnas.2504853122.sapp.pdf]

Supplemental Figures

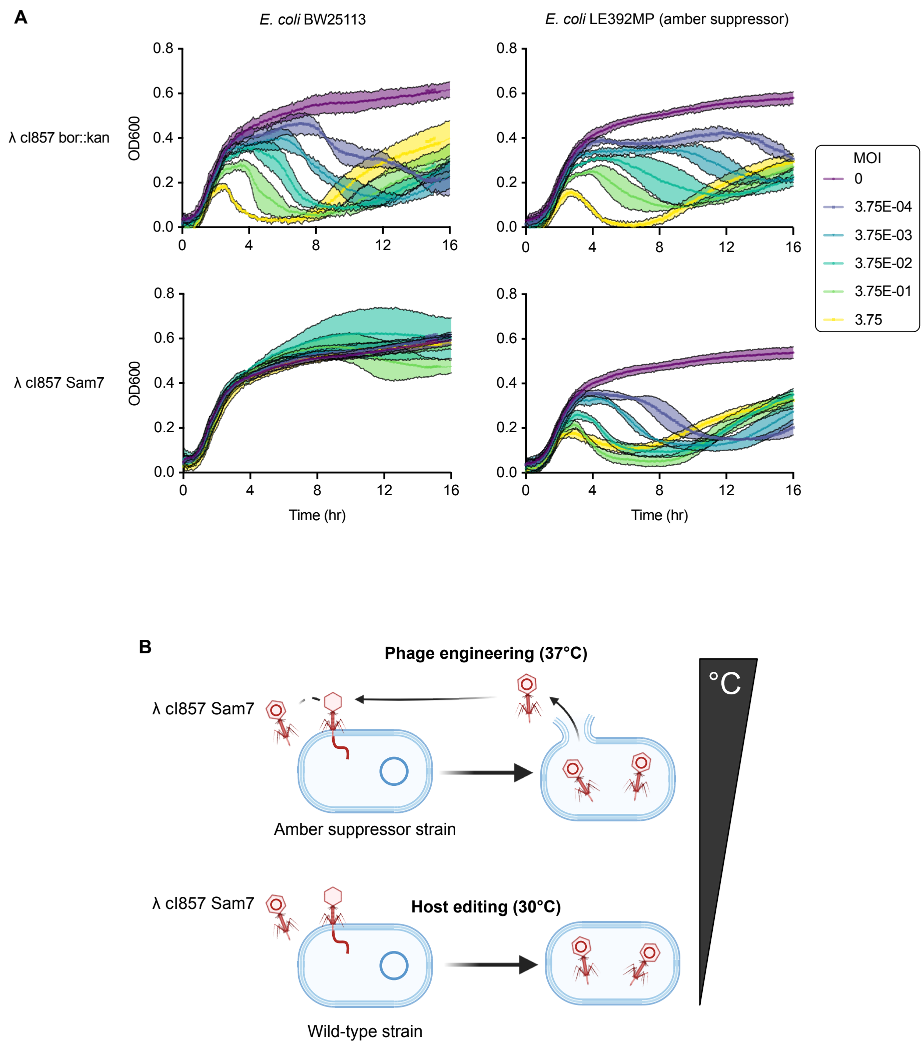

Figure S1 | Effect of host-phage genotypic interactions at different incubation temperatures
(A) *E. coli* strains BW25113 and LE392MP, an amber-suppressor strain, infected by λ cI857 bor::kan or λ cI857 Sam7 at an range MOI of 0-3.75. OD600 was measured over a 16-hour incubation at 37°C with shaking. (B) Graphical representation of the temperature- and host-dependent lysis phenotype of the cI857 Sam7 mutations. At higher temperatures (e.g., 37°C) in an amber-suppressor strain, active lysis occurs, enabling phage engineering. At lower temperatures (e.g., 30°C) in a wild-type host strain, active cell lysis is prevented due to a nonfunctional *S* gene product, enabling phage-mediated host genome editing. Growth curves were plotted using the mean and SD of n = 3 biological replicates.

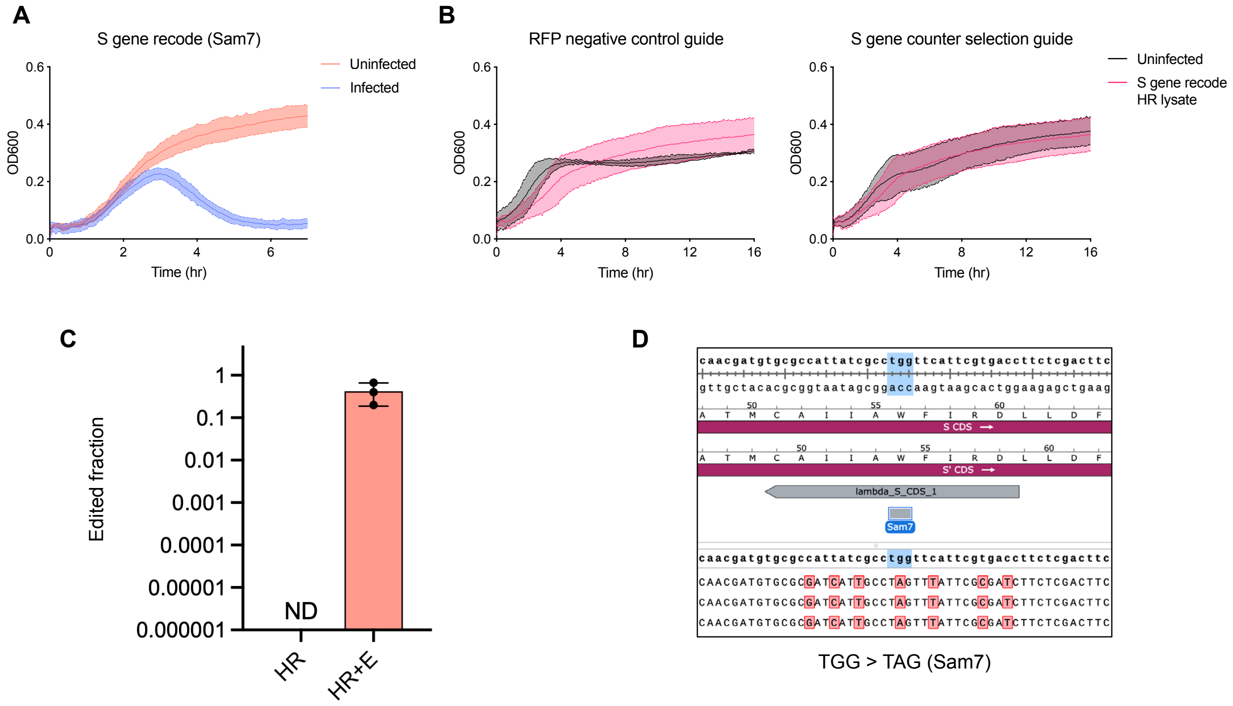

Figure S2 | Recoding of the *S* gene of λ cI857 bor::kan to introduce the Sam7 mutation
(A) Growth over time of a phage editing strain, harboring an HR editing plasmid to recode the *S* gene, infected by λ cI857 bor::kan. (B) Growth over time of counterselection strains, expressing Cas13a with a negative control (RFP target) or counterselection (*S* gene target) guide, infected by the lysate generated from the infected strain in panel A. (C) Edited fraction data of the *S* gene recoding after editing (HR) and subsequent enrichment (HR+E), as determined by plaque assay where phages collected after HR and HR+E were serially diluted and spotted on Cas13a negative control and counterselection strains. (D) Location of the Sam7 recoding edit and Cas13a counterselection guide target in the *S* gene. The Cas13a guide 'lambda_S_CDS_1' targets the corresponding locus of the *S* gene transcript. The codon highlighted in blue (TGG) was edited to introduce the Sam7 mutation (TAG). At the bottom, sequenced PCR products derived from enriched plaques are aligned to the wild-type *S* gene. Bases highlighted in red are edited relative to the wild-type, and silent mutations are present alongside the Sam7 (TGG>TAG) edit to facilitate Cas13a counterselection. Growth curves were plotted using the mean and SD of n = 3 biological replicates.

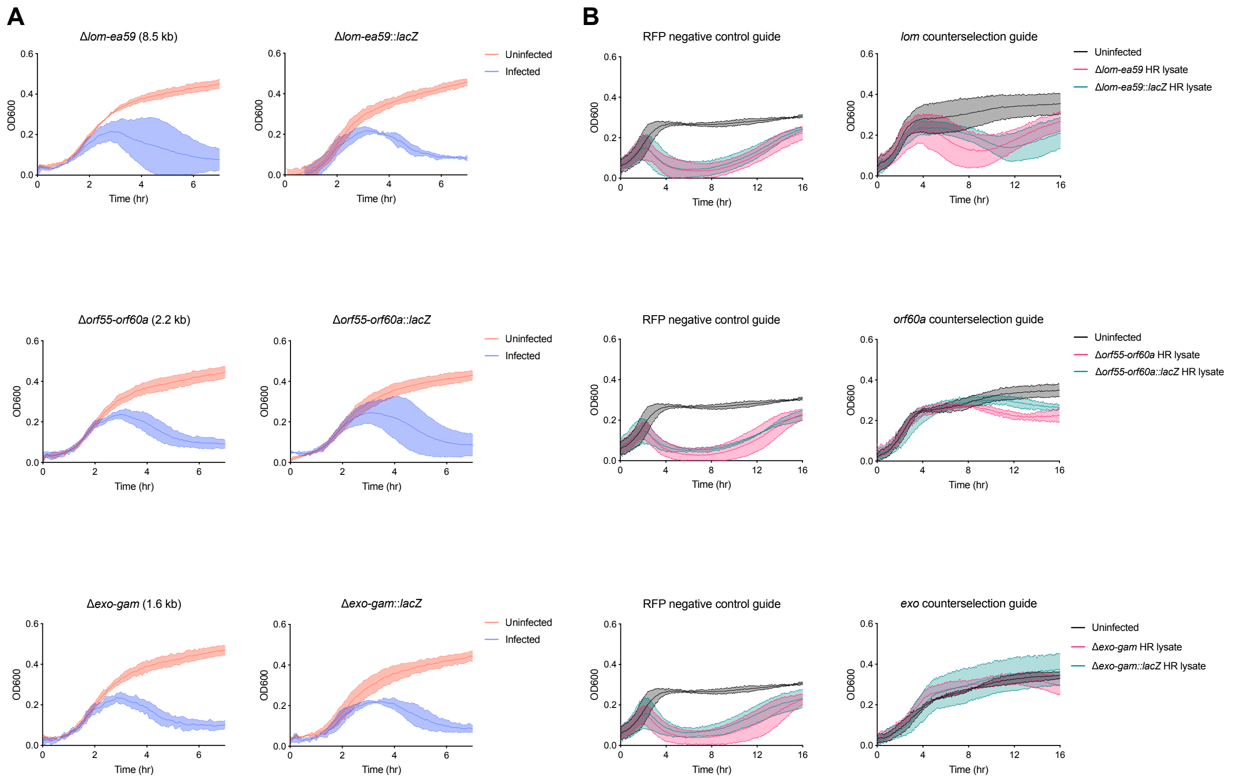

Figure S3 | Growth characteristics of homologous recombination and enrichment steps of λ phage genome editing

(A) Growth over time of various editing strains uninfected or infected by λ cI857 Sam7 bor::kan. (B) Growth over time of Cas13a counterselection strains with a negative control (RFP) or enrichment (*lom*) guide corresponding to edits in part A. For each homologous recombination and enrichment set, phage lysates collected from the homologous recombination step were used to infect corresponding counterselection strains for enrichment of the intended edit. Growth curves were plotted using the mean and SD of *n* = 3 biological replicates.

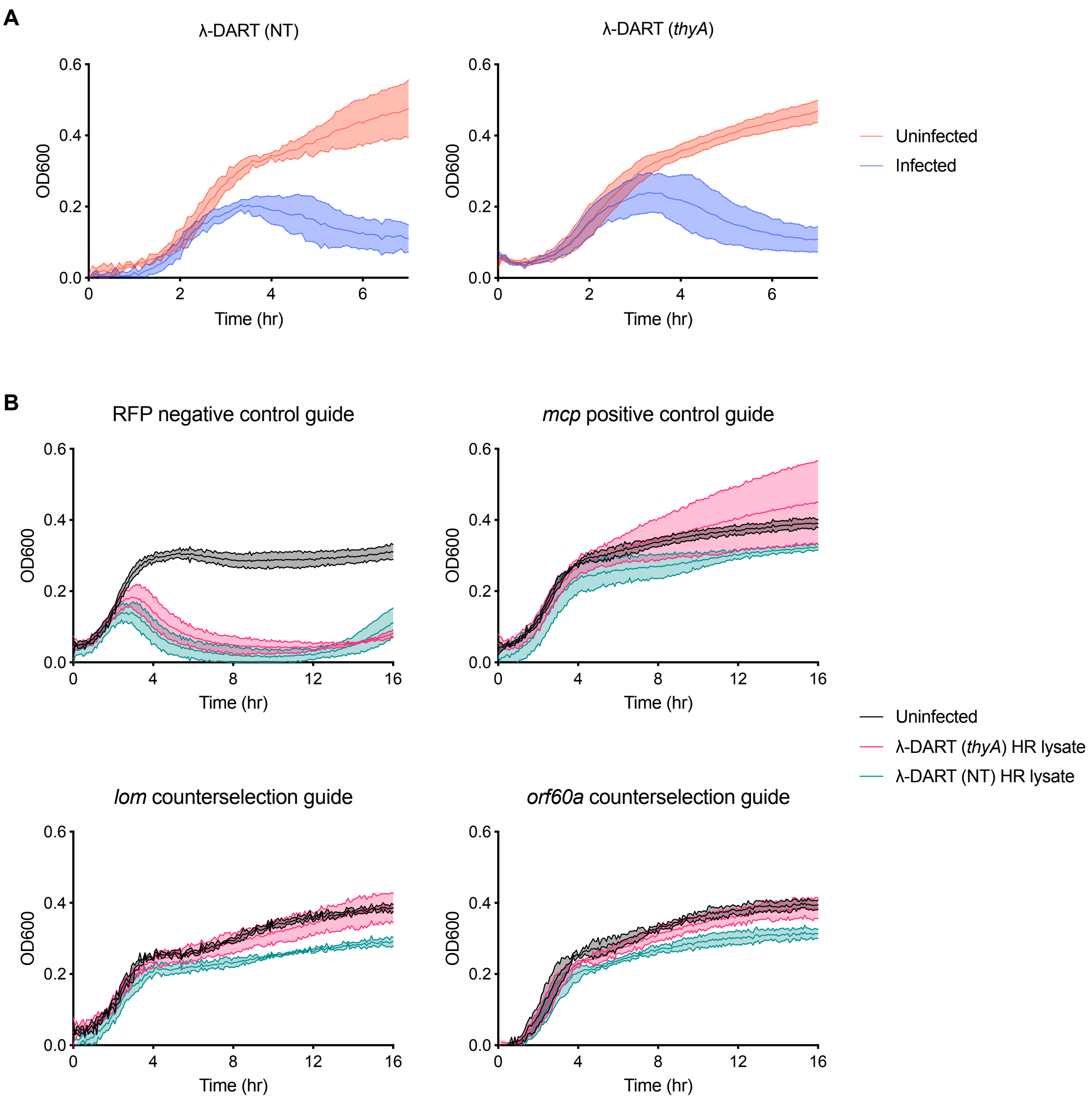

Figure S4 | Representative growth characteristics of phage editing for λ-DART
(A) Growth over time of Δ*lom-orf60a::*DART (NT or *thyA*-targeting DART guides) editing strains infected by λ cI857 Sam7 bor::kan. (B) Growth over time of Cas13a counterselection strains with a negative control (RFP), positive control (*mcp*), or enrichment (*lom* and *orf60a*) guide for the Δ*lom-orf60a::*DART edits in part A. Growth curves were plotted using the mean and SD of *n* = 3 biological replicates.

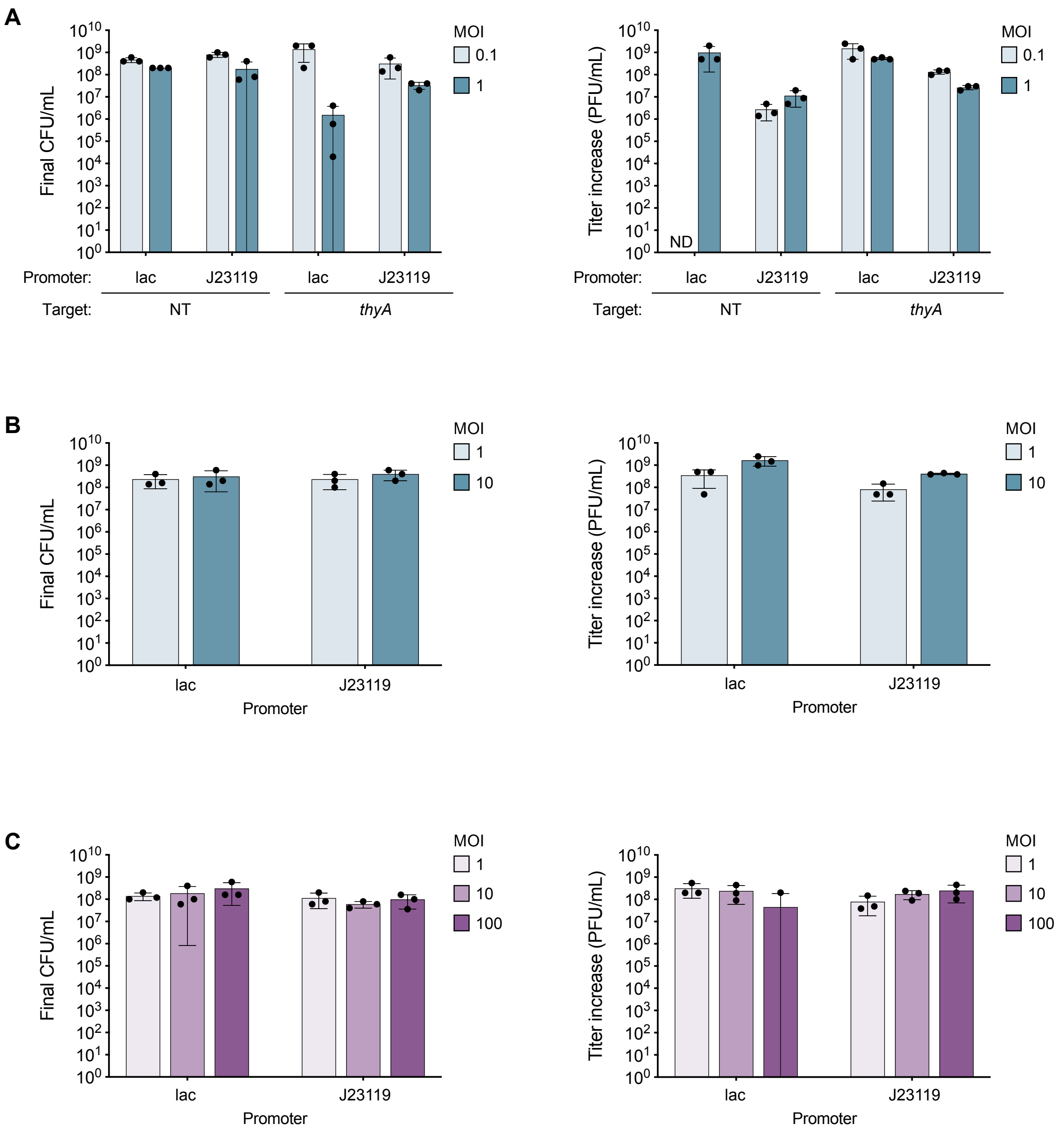

Figure S5 | Colony forming units and phage titers following infection by λ-DART for host genome editing

(A) Colony forming units per mL (CFU/mL) (left) and phage titer increase (right) following the initial 24-hour host genome editing incubation period with the corresponding λ-DART phage and MOI. (B) Colony forming units per mL (CFU/mL) (left) and phage titer increase (right) following the initial 24-hour host genome editing incubation period with the corresponding λ-DART phage and MOI. Panels A and B are associated with Figure 3. (C) Colony forming units per mL (CFU/mL) (left) and phage titer increase (right) following the initial 48-hour host genome editing incubation period with the corresponding λ-DART phage and MOI. Panel C is associated with Figure 5. All plots illustrate the mean, SD, and individual data points from *n* = 3 biological replicates; ND = not detected.

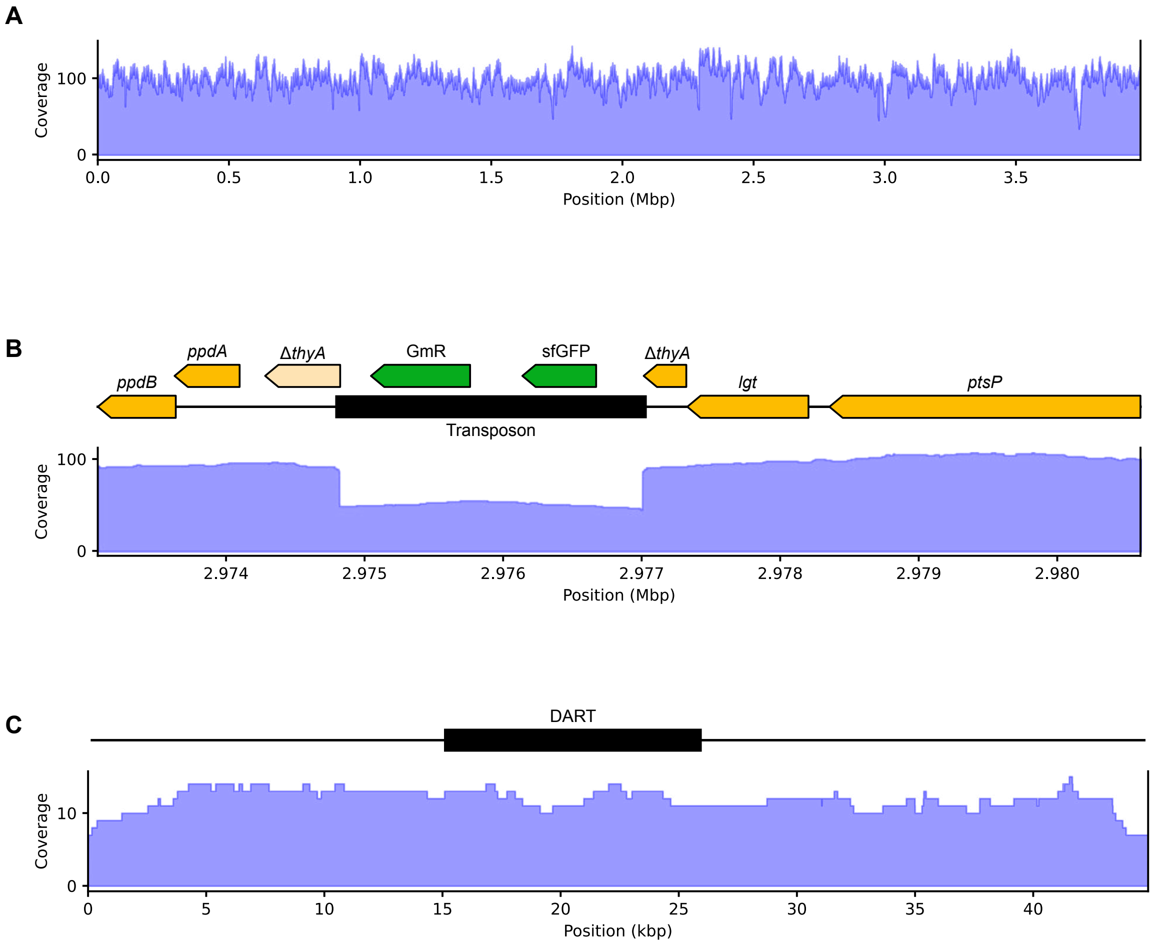

Figure S6 | ONT whole-genome sequencing of a host strain edited by λ-DART

(A) Following infection by λ-DART (*thyA* DART guide), subsequent selective plating for isolated colonies, and confirmatory PCR at the target site, a colony was selected for whole-genome sequencing by Oxford Nanopore long reads. The colony was incubated overnight in liquid culture before DNA extraction occurred. A coverage plot is shown for the 3.974-Mbp chromosome assembled from the reads. (B) A zoom-in of the corresponding region from panel A. The DART transposon is shown integrated at the *thyA* target site, therefore disrupting the gene. (C) A coverage plot for the 44.8-kbp phage assembled from the reads. This assembled phage contained the entire DART edit as designed.

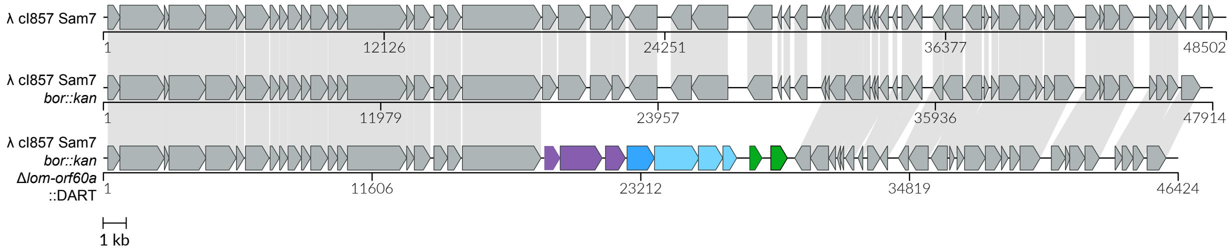

Figure S7 | Whole genome alignment of λ phage genomes of interest

The full genomes of λ and related derivatives, including λ-DART, are illustrated. Homologous proteins are shown as gray bars connecting genes between genomes. Color is applied to λ-DART genes *tnsABC*, *tniQ*, *cas876*, and transposon genes GmR and sfGFP.
